# *CDK12*: A Potent Target and Biomarker for Human Cancer Therapy

**DOI:** 10.3390/cells9061483

**Published:** 2020-06-18

**Authors:** Shujing Liang, Lifang Hu, Zixiang Wu, Zhihao Chen, Shuyu Liu, Xia Xu, Airong Qian

**Affiliations:** 1 Laboratory for Bone Metabolism, Xi’an Key Laboratory of Special Medicine and Health Engineering, Northwestern Polytechnical University, Xi’an 710072, China; liangsj@mail.nwpu.edu.cn (S.L.); hulifang@nwpu.edu.cn (L.H.); wuzx@mail.nwpu.edu.cn (Z.W.); chzhh@mail.nwpu.edu.cn (Z.C.); syliu@mail.nwpu.edu.cn (S.L.); xuxia1036916053@mail.nwpu.edu.cn (X.X.); 2 Key Laboratory for Space Biosciences and Biotechnology, Research Center for Special Medicine and Health Systems Engineering, Northwestern Polytechnical University, Xi’an 710072, China; 3 NPU-UAB Joint Laboratory for Bone Metabolism, School of Life Sciences, Northwestern Polytechnical University, Xi’an 710072, China

**Keywords:** cyclin-dependent kinase 12, gene transcription, cell cycle, cell proliferation, DNA damage response, cancer therapy

## Abstract

Cyclin-dependent kinases (CDKs) are a group of serine/threonine protein kinases and play crucial roles in various cellular processes by regulating cell cycle and gene transcription. Cyclin-dependent kinase 12 (*CDK12*) is an important transcription-associated CDK. It shows versatile roles in regulating gene transcription, RNA splicing, translation, DNA damage response (DDR), cell cycle progression and cell proliferation. Recently, increasing evidence demonstrates the important role of *CDK12* in various human cancers, illustrating it as both a biomarker of cancer and a potential target for cancer therapy. Here, we summarize the current knowledge of *CDK12*, and review the research advances of *CDK12*′s biological functions, especially its role in human cancers and as a potential target and biomarker for cancer therapy.

## 1. Introduction

Cyclin-dependent kinases (CDKs) are a group of serine/threonine protein kinases that are key regulators in various cellular processes [1,2,3]. CDK was first discovered as a cell division cycle (Cdc) gene in yeast [4]. The first cloned Cdc gene was Cdc2, which was named as CDK based on its kinase activity and its role in the cell cycle regulation [5]. With the successive discovery of CDK members, CDKs are divided into two subfamilies, including cell cycle-associated CDKs and transcription-associated CDKs. Cell cycle-associated CDKs mainly contain CDK1, CDK2, CDK4 and CDK6, which directly regulate the cell cycle progression. The transcription-associated CDKs, consisting of CDK7, CDK8, CDK9, CDK11, *CDK12* and CDK13, control gene transcription [6,7]. The activity and substrate specificity of both cell cycle-associated CDKs and transcription-associated CDKs rely on a regulatory subunit known as cyclin. CDK binds a specific cyclin subunit to form a functional and active CDK/cyclin complex [7,8].

*CDK12* is a transcription-associated CDK. It complexes with cyclin K to regulate gene transcription elongation via phosphorylating RNA polymerase II (RNAP II) [9,10,11,12,13] and also regulates translation [14]. Moreover, *CDK12* plays a role in RNA splicing, cell cycle progression, cell proliferation, DNA damage response (DDR) and maintenance of genomic stability [2,9,10,13,14,15,16,17,18]. Since the mutation or amplification of *CDK12* is closely related with tumorigenesis, *CDK12* becomes an attractive therapeutic target for cancer treatment [7,19,20,21]. Here, we introduce the characteristics of *CDK12*, summarize the current advances of its biological functions and highlight its role in human cancer. Furthermore, we also discuss the future research direction of *CDK12*.

## 2. Cyclin Dependent Kinase 12 (*CDK12*): Gene, Structure and Expression

### 2.1. Gene and Isoforms of CDK12

*CDK12*, a ~164 kDa protein consisting of 1490 amino acids, is encoded by **CDK12**, located in human chromosome 17q12 and composed of 14 exons [22]. It was first identified as a novel human protein kinase by Ko et al. from the cDNA of HeLa cell in 2001 [22]. Because it is a Cdc2-related kinase with an arginine/serine-rich (RS) domain, it was named as CrkRS (Cdc2-related kinase with RS domain). Later in 2006, some researchers discovered that cyclin L1 and cyclin L2 were cyclins interacting with CrkRS, and thus CrkRS was renamed as *CDK12* [23]. Chen et al. found that overexpressed *CDK12* complexed with cyclin L via an immunoprecipitation experiment [23]. However, they did not point out the native interaction between *CDK12* and cyclin L. To identify the associations between cyclin and endogenous *CDK12*, Bartkowiak et al. carried out a Mass Spectrometry (MS) analysis on co-immunopurified proteins and found that cyclin K was the only cyclin being identified, which demonstrates that *CDK12* interacts with cyclin K [24]. Subsequent studies also confirmed that the cyclin combining with *CDK12* is cyclin K [8,13,25]. Likewise, CDK13, the homologue of *CDK12*, has been proven to associate with cyclin K [13,25,26,27]. Moreover, cyclin K1 (a ~65 kDa isoform of cyclin K) has been demonstrated as the primary cyclin partner for *CDK12* [8,13,25].

**Figure 1:**
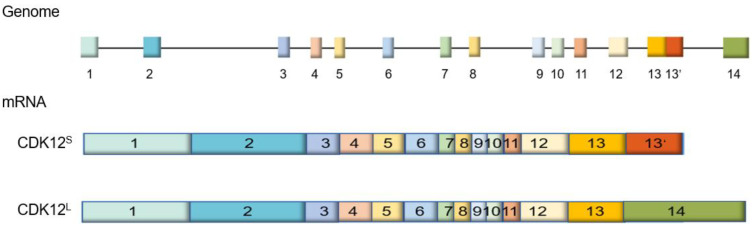
Genomic and messenger RNA (mRNA) structures of cyclin-dependent kinase 12 (*CDK12*). *CDK12*^S^: the shorter isoform of *CDK12*, *CDK12*^L^: the longer isoform of *CDK12*.

There are two isoforms of *CDK12*, which are identical at the 5′ end but different at the 3′ end [23] (Figure 1). According to the length of the open reading frame, the two *CDK12* isoforms are named as *CDK12*^S^ (the shorter isoform of *CDK12*) and *CDK12*^L^ (the longer isoform of *CDK12*), respectively [23] (Figure 1).

### 2.2. Structure of CDK12

**Figure 2:**
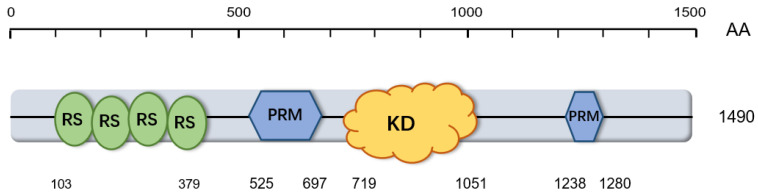
Schematic diagram of *CDK12* protein structure. AA: amino acid; RS: arginine/serine-rich domain; PRM: proline-rich motif; KD: kinase domain.

*CDK12* is mainly composed of three domains: a central Cdc2-related protein kinase domain (KD), an *N*-terminal “arm”, about 700 amino acids, and a *C*-terminal “arm”, about 500 amino acids [22,28] (Figure 2). The central KD is composed of 300 amino acids and is located at the center of *CDK12* [22]. Its main function is to mediate the phosphorylation of the *C*-terminal domain (CTD) of RNAP II. There are 21 RS motifs in the first 400 amino acids of *CDK12*, and only one RS motif in the rest of the approximately 1000 amino acids [22]. The RS domain, which is enriched arginine and serine, is considered as a prominent feature of *CDK12* [22]. It was originally found in pre-messenger RNA (pre-mRNA) splicing factors that were important for spliceosome assembly and alternative splice-site selection [29]. In *CDK12*, the RS domain mainly functions to target *CDK12* to the nuclear speckles [22]. The central KD and the RS domain endow *CDK12* the capacity to directly link transcription with the splicing machinery. Proline-rich motifs (PRM) are located between the RS domain and the central KD and are also found in the *C*-terminal region [22]. The PRM contains the consensus binding sites for Src homology 3 (SH3) and tryptophan (WW) regions which can mediate protein–protein interactions by binding proline-rich modules in ligands [30,31,32,33,34,35]. The presence of the RS domain and PRM domain indicates that *CDK12* is likely to take part in numerous protein–protein interactions [28]. Notably, the closest human homologue of *CDK12* is CDK13. While their sequences of KD are highly homologous, their *C*- and *N*-terminal regions differ between *CDK12* and CDK13 [26,28].

### 2.3. CDK12 Expression

As a transcription-associated CDK, *CDK12* is ubiquitously expressed in mammalian tissues. The presence of *CDK12* in all tissues has been determined via screening a panel of RNAs from specific human tissues [22]. *CDK12* shows low tissue specificity according to The Human Protein Atlas (available online: https://www.proteinatlas.org/). Notably, high expression of *CDK12* has been observed in bone marrow and testis compared with other tissues by The Human Protein Atlas. Besides, Castillo et al. have experimentally confirmed the high expression of *CDK12* in human testis [36].

## 3. *CDK12*′s Biological Functions

### 3.1. CDK12 in Gene Transcription

In 2010, *CDK12* was first demonstrated as a transcription-associated CTD kinase in *Drosophila* [24]. At present, *CDK12* is regarded as a transcription-associated CDK, which phosphorylates the CTD of RNAP II [8,9,24,37]. RNAP II is responsible for RNA synthesis of eukaryotic genes. It directs the gene transcription process consisting of transcription initiation, elongation and termination [38]. The large subunit of RNAP II is RPB1 which contains a CTD. CTD contains repeats of the heptapeptide Y_1_S_2_P_3_T_4_S_5_P_6_S_7_, and single serine phosphorylation in these repeats is required for each step of the transcription cycle [39]. Phosphorylation of Ser2 is a hallmark of transcription elongation, and phosphorylation of Ser5 is required for proper transcription initiation, both of which are necessary for the transcription cycle [38,40]. Bartkowiak et al. have shown that treatment with RNA interference (RNAi) of *CDK12* alters the phosphorylation state of the CTD and reduces the phosphorylation level of Ser2 [24]. Other findings have also found that *CDK12* predominantly phosphorylates Ser2 [8,12,13,37,41,42]. Therefore, *CDK12* is considered to phosphorylate Ser2 but not Ser5. In addition, *CDK12* and cyclin K are considered to be proteins associated with RNAP II and transcription elongation [24,43]. *CDK12* binds cyclin K to form a *CDK12*/cyclin K complex, which regulates phosphorylation of Ser2 in the CTD of RNAP II and expression of DDR genes, DNA replication genes and DNA repair genes [9,13].

Interestingly, RNAP II transcription is not globally impaired in cells without *CDK12*/cyclin K complex [13]. Chirackal Manavalan et al. have found that the inhibition of *CDK12* does not affect Ser2 phosphorylation level as well as global transcription but diminishes RNAP II processivity accompanied by transcript shortening of DNA replication genes, which is consistent with defective transcription elongation [9]. Moreover, *CDK12* also plays a role in co-transcriptional processing of genes such as *MYC,* particularly at its 3′ end [41]. The Ser2 phosphorylation of *CDK12* is important for the recruitment of 3′ end formation factors like cleavage stimulation factor 77 (CstF77). This mechanism involves RNAP II pausing that promotes Ser2 phosphorylation of *CDK12*, which serves to recruit CstF77 and is necessary for optimal 3′ end processing of the *MYC* gene [41]. Similarly, *CDK12* is required for 3′ end processing of cellular oncogene fos (c-FOS) transcripts. Depletion of *CDK12* leads to decreased levels of Ser2 phosphorylation, cleavage stimulation factor 64 (CstF64) and cleavage, and polyadenylation specificity factor 73 (CPSF73) at the *c-FOS* gene and attenuates the 3′ end formation of c-FOS transcripts [44]. In summary, *CDK12* plays a key role in gene transcription.

### 3.2. CDK12 in RNA Splicing

*CDK12* has been shown to play a role in RNA splicing. Rodrigues et al. have identified *Drosophila* *CDK12* as a major determinant in regulating HOW (held out wings, a RNA-binding protein)-dependent splicing of Neurexin IV (a cell-adhesion molecule) [45]. Thus, they have demonstrated a mechanism in regulating timed splicing of newly synthesized mRNA molecules through phosphorylating of RNAP II CTD [45]. In addition, *CDK12* is proven to alter splicing site selection of an E1a minigene [23]. Depletion of *CDK12* shows diminished 3′ end processing of the activated *c-FOS* and *c-MYC* genes [41,44]. These findings demonstrate the importance of *CDK12* in regulating and coordinating the transcription and pre-mRNA processing. Furthermore, *CDK12* stabilizes serine-arginine splicing factor 1 (SRSF1) mRNA transcripts through skipping an alternative intron in the 3′ untranslated region (3′ UTR) [46]. Moreover, *CDK12* associates with core spliceosome components and regulates alternative last exon (ALE) splicing of long transcripts in various cell types [20].

Recent studies have shown that minimal splicing alterations induced by the inhibition of *CDK12* may be due to defective transcription elongation [9,10]. Treatment with THZ531 (inhibitor of *CDK12*) results in 13.4% intron retention, which is the largest proportion of splicing alteration [10]. Importantly, this phenomenon occurs primarily in long genes, such as DDR genes [10]. The apparent increased splicing efficiency in long genes may be due to defective transcription elongation accompanied by the reduction in the formation of such long transcripts, rather than a more efficient spliceosome [10]. Moreover, as DDR genes contain more intronic polyadenylation sites than other expressed genes, *CDK12* can regulate DDR genes via suppressing the intronic polyadenylation [11]. It is worth noting that inhibition of *CDK12* leads to transcript shortening of genes, which affects the expression of DNA replication genes and DNA repair genes [9]. In addition, the defective RNAP II processivity is usually accompanied by slower transcription elongation rates of *CDK12*-sensitive genes [9]. Thus, all these findings indicate that *CDK12* indirectly regulates RNA splicing through regulating gene transcription.

### 3.3. CDK12 in Translation

Besides the regulatory role in mRNA biosynthesis, *CDK12* also regulates the translation of mRNA [14]. Choi et al. found that *CDK12* promoted translation of mRNAs via phosphorylating 4E-binding Protein 1 (4E-BP1), the mRNA 5′ cap-binding repressor [14]. More specifically, *CDK12* cooperates with the mechanistic target of rapamycin (mTORC1) to affect the translation of mRNAs encoding DNA repair factors, ribosome and translation factors via phosphorylating 4E-BP1 at two Ser–Pro sites (S65, T70) that control the exchange of 4E-BP1, with eukaryotic initiation factor 4G (eIF4G) at the 5′ cap of target mRNAs. This finding reveals a new set of target genes (mTORC1-regulated genes) of *CDK12*. Therefore, *CDK12* is important in the correct arrangement and progression of chromosomes through mitosis [14].

### 3.4. CDK12 in Cell Cycle

Normal cell cycle progression is of great significance for cell proliferation and the maintenance of genomic stability [9,47]. The dysregulation of cell cycle progression contributes to abnormal cell proliferation and oncogenesis [48,49]. The progression of the cell cycle relies on the periodic activity of the complex that binds CDKs and cyclins [50]. Different CDK/cyclin complexes exhibit different CDK kinase activity, thereby affecting different stages of cell cycle [50].

*CDK12* plays a role in regulating cell cycle progression. Long-term depletion of *CDK12* induces cell accumulation in G2/M phase [13,51]. Chen et al. conditionally deleted *CDK12* in the neural progenitor cells (NPCs) of mice and found that the NPCs were accumulated at G2 and M phase [52]. There was a 1.3–4.6-fold increase of mitotic cells in the mutant mice compared with the control mice, suggesting that the cells lacking *CDK12* had a longer cell cycle [52]. Therefore, deletion of *CDK12* has been shown to prolong cell cycle, indicating the role of *CDK12* in regulating cell cycle. A recent study has demonstrated that knockdown of *CDK12* or cyclin K results in induction of mitotic catastrophe and decreased expression of Aurora B, a key regulator of mitosis [53]. The depletion of cyclin K induces inhibition of proliferation accompanied by G2/M arrest [53]. More recently, Chirackal Manavalan et al. have found that inhibition of *CDK12* induces the G1/S cell cycle progression defect by using an analog-sensitive *CDK12* cell line, in which *CDK12* can be rapidly and specifically inhibited [9]. Inhibition of *CDK12* induces the decreased expression of some crucial DNA replication genes (e.g., *TOPBP1* (DNA topoisomerase II binding protein 1), *CDC6* (cell division cycle 6) and *CDT1* (Cdc10-dependent transcript 1)), which disrupts the formation of pre-replicative complex (pre-RC), thereby delaying G1/S progression [9]. This illustrates that *CDK12* controls G1/S progression by regulating the expression of core DNA replication genes [9]. Moreover, the treatment of RNAi of *CDK12* significantly increases the cell number of G0/G1 phase, indicating that *CDK12* plays an important role in controlling the transition of G0/G1 phase to S phase [54].

### 3.5. CDK12 in Cell Proliferation

As cell cycle is closely related with cell proliferation, aberrant cell cycle progression may result in abnormal cell proliferation. *CDK12* is involved in cell proliferation by regulating cell cycle. The assembly of pre-RC occurs during G1 phase, a process referred to as replication origin licensing, which is indispensable for sustaining cell proliferation [55]. Knockdown of cyclin K or its cognate kinase *CDK12* prevents the assembly of pre-RC in G1 phase and inhibits cell proliferation [16], suggesting the involvement of *CDK12* in cell proliferation. *CDK12*/cyclin K deficiency has been shown to inhibit cell proliferation and induce apoptosis via the induction of mitotic catastrophe [53]. Moreover, Choi et al. have found that *CDK12*/cyclin K complex is required for multiple steps in mitosis [14]. Cells deficient in *CDK12* or cyclin K display profound mitotic defects [14]. Recently, it was found that a high level of *CDK12* in various human cancers characterized by uncontrolled cell proliferation indicates the important regulatory role of *CDK12* in cell proliferation [16]. Zhang et al. firstly used the *CDK12* inhibitor, THZ531, to treat leukemia cells and found that THZ531 treatment caused an irreversible decrease in cell proliferation [12]. Subsequent studies also reported that suppression of *CDK12* with either short hairpin RNAs (shRNAs) or THZ531 strongly inhibited cell proliferation and impaired the colony formation in cancer cells [51,56].

### 3.6. CDK12 in DNA Damage Response (DDR)

DDR is biologically significant because it is responsible for detecting the DNA damage and repairing it to maintain normal cellular processes [57]. The *CDK12*/cyclin K complex plays an important role in regulating the expression of DDR genes by phosphorylating RNAP II CTD [9,13]. Inhibition of *CDK12*/cyclin K results in decreased expression of DDR genes, such as *BRCA1* (breast and ovarian cancer type 1 susceptibility protein 1), *ATR* (ataxia telangiectasia and Rad3-related), *FANCI* (Fanconi anemia complementation groups - I) and *FANCD2* (Fanconi anemia complementation groups - D2) [13], which are important for maintaining genome stability. Moreover, cells without *CDK12*/cyclin K are sensitive to DNA damage agents and develop spontaneous DNA damage signaling [13]. Therefore, *CDK12* is important for maintaining genomic stability by interacting with cyclin K to regulate the expression of DDR genes [13]. In addition, *CDK12* is proven to regulate pre-RC assembly during G1 phase as well as the expression of DNA replication genes and homologous recombination (HR) DNA repair genes to protect cells from genomic instability [9,16]. Zhang et al. have shown that after the cells are treated with THZ531 (*CDK12* inhibitor), the expression of core DDR genes (*BRCA1*, *FANCF* (Fanconi anemia complementation group F) and *ERCC4* (excision repair cross-complementing group 4)) is decreased [12]. 

The molecular basis for the effect of *CDK12* on DDR genes has been further studied [10]. It has been indicated that inhibition of *CDK12* leads to a gene length-dependent elongation defect associated with early termination through premature cleavage and polyadenylation (PCPA). Thus, the expression of DDR genes is affected by *CDK12*, primarily due to their relatively longer length and lower ratio of U1 small nuclear ribonucleoprotein (U1 snRNP) binding to intronic polyadenylation site [10]. Chirackal et al. have also confirmed that *CDK12* is essential for optimal RNAP II processivity at longer genes, such as genes involved in DNA replication and DNA repair [9]. Recently, it has been reported that *CDK12* responds to DNA damage through regulating the translation of mTORC1-dependent mRNAs [14]. To be more specific, *CDK12* regulates translation of the DNA damage response checkpoint kinase 1 (CHK1). Therefore, *CDK12* acts indirectly to control p53 stability in response to DNA damage through regulating the translation of CHK1 [14]. In addition, *CDK12* selectively regulates the translation of many critical mitotic regulatory complexes. Loss of *CDK12* results in defective DNA repair, mitotic catastrophe and profound genome instability [14,53].

## 4. *CDK12* and Human Cancer

Recently, more and more evidence demonstrates the involvement of *CDK12* in cancer (Table 1) [7,10,15]. This may be due to the key role of *CDK12* in regulating transcription elongation and the expression of genes involved in DDR, DNA replication and mRNA processing [9,10,12,24]. Abnormal expression or mutation of *CDK12* is detected in various cancers, such as breast cancer, ovarian cancer, prostate cancer and gastric cancer [7,15,17,18]. Moreover, *CDK12* is also indirectly implicated in esophageal, endometrial, uterine, bladder, colorectal and pancreatic ductal carcinomas [15]. Interestingly, *CDK12* shows both tumorigenic and tumor-suppressive effects in different cancer types, which will be introduced in detail in the following.

### 4.1. CDK12 in Breast Cancer

Increasing evidence shows that *CDK12* is closely linked with breast cancer. Interestingly, *CDK12* plays distinguishing roles among various subtypes of breast cancer, especially for HER2 (human epidermal growth factor receptor 2)-positive breast cancer and triple-negative breast cancer (TNBC). In HER2-positive breast cancer, *CDK12* acts as a tumor promoter, while in TNBC, *CDK12* acts as a tumor suppressor.

HER2-positive breast cancer is a subtype of breast cancer and presents an amplification pattern of oncogene *HER2* (*ERBB2*). It is shown that *CDK12* and *HER2* oncogenes are co-amplified in breast cancer [20]. *CDK12* promotes migration and invasion of HER2-positive breast tumor cells through regulating the ALE splicing of DDR activator ATM (ataxia telangiectasia-mutated) and DNAJB6 (DnaJ homolog subfamily B member 6, MRJ)-L [20]. In addition, Chen et al. have demonstrated that mutations in *CDK12*, TP53 (tumor suppressor p53) and PIK3CA are the most frequent in 107 HER2-positive breast cancer patients [58]. Choi et al. have indicated that *CDK12* drives the development of HER2-positive breast cancer via affecting WNT (Wingless-Integrated) and IRS1 (insulin receptor substrate-1)-ErbB (epidermal growth factor receptor)-PI3K (phosphatidylinositol-3-kinase) signaling [59]. *CDK12* promotes tumor initiation through regulating cancer stem cells (CSCs) or affecting the genes which are necessary to activate downstream pathways such as ErbB-PI3K-AKT (Protein Kinase B) or WNT-signaling cascades [59]. In addition, inhibition of *CDK12* facilitates anticancer efficacy of trastuzumab in HER2-positive tumors [59]. Another subtype of breast cancer is TNBC, which can be characterized by the low expression of estrogen receptor (ER), progesterone receptor (PR) and HER2 [60]. High expression of *CDK12* is associated with HER2 status and plays important roles during the tumorigenesis and development of breast cancer [61]. However, *CDK12* is not an independent predictor of breast cancer-specific survival [61]. Notably, absent *CDK12* is associated with a triple-negative phenotype (ER-, PR-, HER2-) [61]. There is a small proportion of HER2-positive patients that show absent *CDK12* protein expression but a large proportion of absent *CDK12* protein expression in TNBC patients [61]. In addition, absence of *CDK12* protein is often accompanied by downregulation of DDR proteins (ATR (ataxia-telangiectasia and Rad3-related), Ku70/Ku80 (the classical non-homologous end joining (cNHEJ) factors), PARP1 (poly ADP-ribose polymerase 1), DNA-PK (DNA-dependent protein kinase) and γH2AX (phosphorylated histone H2AX)), suggesting a novel mechanism of *CDK12*-associated DDR dysregulation in breast cancer [61]. In summary, *CDK12* acts as tumor promoter in HER2-positive breast cancer, but as a tumor suppressor in TNBC.

### 4.2. CDK12 in Ovarian Cancer

Ovarian cancer is one of the most common malignant tumors for women. High-grade serous ovarian cancer (HGSOC) has a higher mortality rate [62]. The mutations of TP53 play a dominant role in HGSOC and mutated BRCA1/BRCA2 are found in 22% of tumors [63]. Significantly, the mutation of CDK12 is detected, which is mainly nonsense or indel, suggesting the potential loss of function [63]. Loss-of-function (LOF) mutations of CDK12 contribute to genomic instability, underlying the genesis of the cancer by causing defects in multiple DNA repair signaling pathways [21]. More importantly, *CDK12* LOF genomic alterations are associated with focal tandem duplications (FTDs) in ovarian cancer [64]. In addition, *BRCA1* promoter hypermethylation or mutational inactivation of CDK12 can downregulate transcription of *BRCA1*, thereby disrupting HR DNA repair in ovarian cancer, and then leading to metabolic reprogramming of ovarian cancer cells [65]. A c.1047-2A>G splice site variant of the *CDK12* gene was recently reported to be strongly associated with hereditary ovarian cancer [66]. These results demonstrate that CDK12 is a tumor suppressor in ovarian cancer. Moreover, recent reports have shown that suppression of MYC via inhibition of CDK7, CDK12 and CDK13 may be an effective treatment for MYC-dependent ovarian cancer [67].

### 4.3. CDK12 in Prostate Cancer

Prostate cancer (PCa) is the second most frequently diagnosed cancer in men. It was recently demonstrated that *CDK12* is associated with PCa [68,69]. It is considered that loss or mutation of *CDK12* leads to genomic instability, which contributes to metastatic prostate cancer [68]. In addition, *TP53*, *PTEN* (phosphatase and tensin homolog) and *CDK12* defects are commonly detected in metastatic castration-resistant prostate cancer (mCRPC) patients [70]. It has been shown that inactivation of *CDK12*, TP53 and *BRCA2* affects distinct classes of structural variation in mCRPC based on a whole-genome analysis from 101 mCRPC patients [71]. Specifically, *CDK12* mutation is related to tandem duplications [71]. Recent studies also show that inactivation of *CDK12* (biallelic inactivation) is associated with a global tandem duplication phenotype [72,73]. Wu et al. have identified a novel subtype of prostate cancer characterized by biallelic loss of *CDK12* [69]. They detected the aberrations of *CDK12* in 25/360 mCRPC patients (6.9%). In addition, the *CDK12* mutant is often accompanied by FTDs [64,69]. The presence of FTDs in *CDK12*-mutated cancers may result in highly recurrent gains of genes involved in cell cycle and DNA replication [69]. In addition, *CDK12*-mutant prostate cancers are characterized by increased gene fusions, fusion-induced neoantigen open reading frames and high immune infiltration [69]. *CDK12*-mutant prostate cancer patients have a higher likelihood of response to immunotherapy than an unselected metastatic prostate cancer population from the pilot clinical study [69]. Therefore, inhibition of *CDK12* may sensitize tumors to checkpoint inhibitor-based immunotherapies [69].

### 4.4. CDK12 in Gastric Cancer

*CDK12* is also involved in gastric cancer. According to the different status of HER2, gastric cancer is divided into two subtypes, including HER2-positive gastric cancer and HER2-negative gastric cancer [74]. Among these, *CDK12* amplification is mainly detected in HER2-positive gastric cancer [74]. Ji et al. have shown that high-level expression of *CDK12* is detected in gastric tumor samples compared with normal samples [75]. Moreover, patients with high expression of *CDK12* show lower overall survival rates than patients with low expression of *CDK12* [75]. They have identified positive correlations of CD8+ cell number and CCL21 (CC-chemokine ligand 21) mRNA expression with *CDK12* level [75]. These evidences indicate the involvement of *CDK12* in gastric cancer.

## 5. *CDK12* as a Potential Target and Biomarker for Cancer Therapy

Evidence shows that *CDK12* is not only a biomarker but also a potential therapeutic target of cancer (Table 2). *CDK12* mutation or deficiency sensitizes cells to PARP (poly ADP-ribose polymerase) inhibitors and agents that target cell-cycle checkpoints, such as CHK1 [77]. PARP is a nuclear enzyme that modifies the substrates by poly(ADP-ribose)ylation (PARylation) [78]. PARP inhibitors are Food and Drug Administration (FDA)-approved drugs that target cancers with defects in HR, including those with *BRCA1* or *BRCA2* mutations [79,80]. Cancers with a *BRCA1* mutation, such as TNBC and ovarian cancer, are usually treated with PARP inhibitors as targeted drugs [79]. Johnson et al. have indicated that loss or inhibition of *CDK12* sensitizes cells to PARP inhibitors and helps patients overcome the resistance of PARP inhibitors [81]. CHK1 is a cellular factor that targets tumor cells with genomic instability [82]. CHK1 inhibitors have been tested as anti-tumor agents and are used in treating a variety of cancers [79]. Loss of *CDK12* enhances the anti-proliferative effect of CHK1 inhibitors [79]. Previous studies have shown that the anti-tumor effect of CHK1 inhibitors is determined by p53 status, while other findings have illustrated that CHK1 inhibitors decrease cellular viability irrespective of p53 status [83,84,85]. However, Paculova et al. have indicated that the anti-proliferative effect of CHK1 inhibitor combined with loss of *CDK12* is comparable in cell lines regardless of p53 status [79]. CHK1 is important in the effective repair of endogenous DNA damage, especially in cells lacking *CDK12* or BRCA1 [79]. Thus, *CDK12* deficiency should be considered as a CHK1 sensitivity biomarker candidate [79].

*CDK12* plays an important role in promoting cancer cell growth, especially in cancers driven by dysregulated transcription factors, such as cancers dependent on MYC (neuroblastoma) and the EWS–FLI1 fusion oncoprotein (Ewing sarcoma) [77]. Neuroblastoma is a cancer highly dependent on transcriptional programs [86]. *MYC* is a proto-oncogene and a major driver of many human cancers. Amplification of *n-MYC* (*MYCN*) leads to neuroblastoma [87]. Studies have indicated that THZ1, a *CDK12* inhibitor, inhibits MYC expression and tumor growth [67,87]. In addition, *CDK12* plays an important role in the processing of MYC precursor mRNA. Ewing sarcoma is characterized by chromosome rearrangement which fuses the strong transactivation domain of EWS protein with the DNA binding domain of FLI1 protein [86]. EWS/FLI acts as both a transcriptional activator and a transcriptional repressor [88]. Currently, treatment of Ewing sarcoma mainly uses *CDK12* inhibitors THZ1 and THZ531, which impair DNA damage repair in an EWS/FLI-dependent manner [86]. The combination of *CDK12* and PARP inhibitors is highly active in Ewing Sarcoma [86]. Taken together, targeting *CDK12* may be a viable treatment strategy for cancers driven by dysregulated transcription factors.

CDK inhibitors have been studied and applied to cancer treatment. Considering that *CDK12* plays an important role in regulating transcription elongation and maintenance of genome stability, *CDK12* aberrations are found in various types of cancer [59,61,63,69]. Inhibition of *CDK12* is considered a favorable strategy for cancer treatment [24,81,89,90,91]. Dinaciclib is a multi-specific CDK inhibitor that exhibits potent antiproliferative effects on various cancers [92]. It was initially found to inhibit CDK1, CDK2, CDK5 and CDK9, and was recently reported to have an inhibitory effect on *CDK12* [81,93]. Dinaciclib inhibits phosphorylation of Ser2 of RNAP II CTD and downregulates HR DNA repair genes [81]. Moreover, dinaciclib can reverse the resistance of PARP inhibitor, converting tumor growth inhibition to durable regression [81]. This suggests that combined inhibition of *CDK12* and PARP may be a good therapeutic strategy. THZ1 is a CDK7 inhibitor and has therapeutic effects on both breast and lung cancer [94,95]. Currently, it has been demonstrated that high concentrations of THZ1 can also be used as an inhibitor of *CDK12* [90]. Based on the study of THZ1, THZ531 (a novel *CDK12* inhibitor) is developed [12]. In 2016, Zhang et al. designed a covalent inhibitor, THZ531, which can inhibit both *CDK12* and CDK13 [12]. Studies have indicated that THZ531 inhibits cell proliferation via preferentially suppressing the expression of DNA repair-related genes and inducing strong DDR in cancer cells [56]. Another novel *CDK12*/13 inhibitor, SR-4835, has been developed by Quereda’s group [76]. It has potential for treatment of TNBC through the downregulation of core DDR genes and upregulation of genes involved in cell apoptosis. Accordingly, SR-4835 is synergized with PARP inhibitors to inhibit cancer cell proliferation [76]. Clinical trials of *CDK12* inhibitors combined with PARP inhibitors treatment are currently under way. One clinical trial in Phase I adopts dinaciclib (CDK inhibitor SCH 727965) and veliparib (PARP-1 inhibitor ABT-888) for treatment in patients with advanced solid tumors (available online: http://clinicaltrials.gov, NCT01434316). This clinical trial is still in the process of recruiting and is estimated to be completed in December 2020. Once the recommended phase 2 dose for ABT-888 in combination with SCH727965 is established, the trial will be included in an extended cohort to assess preliminary activity in both *BRCA* wild-type and *BRCA*-mutated TNBCs. In summary, *CDK12* inhibitors may become good candidates for anticancer drugs.

## 6. Conclusion and Perspectives

Here, we summarized the current knowledge and research advances of *CDK12* and its biological functions, and highlighted the role of *CDK12* in human cancers, demonstrating that it is a potential target for cancer therapy. By regulating transcription elongation and the expression of genes involved in DDR, DNA replication and mRNA processing, *CDK12* participates in various cellular processes such as DDR, RNA splicing, cell cycle progression and cell proliferation. As all of these cellular processes are closely related to cancer development, *CDK12* has been demonstrated as an important molecule involved in cancer development, such as breast cancer, ovarian cancer, prostate cancer and gastric cancer. This suggests that *CDK12* is an important biomarker and may serve as a potential therapeutic target. More recent studies have verified the therapeutic effects by targeting *CDK12* with *CDK12* inhibitors during the treatment of cancers. The *CDK12* inhibitors not only inhibit the transcription and proliferation of cancer cells but also enhance the sensitivity of tumor cells to drugs and overcome drug resistance. Moreover, suppression of *CDK12* has a good therapeutic effect on cancers, especially those driven by dysregulated transcription factors. Recently, *CDK12* inhibitors have been applied for clinical trials for cancer treatment. All these demonstrate *CDK12* as a biomarker and target for cancer diagnosis and therapy.

In summary, *CDK12*, a transcription-associated CDK, shows versatility in regulating gene transcription, RNA splicing, translation, cell cycle, cell proliferation and DDR, alteration of which contributes to cancer development. Thus, the alteration of *CDK12* drives tumorigenesis. Given their biological functions, important roles among various cancers, and the therapeutic effects for cancer cells by targeting *CDK12*, *CDK12* may become a novel potential biomarker and target for human cancer diagnosis and therapy in the future. However, there are still some concerns. As *CDK12* is important for normal cell cycle progress and cell proliferation, will the modulation of *CDK12* when treating cancer induce other disease? How can we specifically target *CDK12* in the cancer cells? Considering the complicated role of *CDK12* acting both as a tumor suppressor and promoter, especially for different subtypes of breast cancer, what kind of strategy should be adopted to target *CDK12* for each subtype (e.g., triple-negative breast cancer)? As some *CDK12* inhibitors also target CDK13, how should these inhibitors be used for clinical application and how do we develop the inhibitor specifically targeting *CDK12*? Answering these questions will make *CDK12* a potential target for cancer therapy.

## Figures and Tables

**Table 1:** The role of cyclin-dependent kinase 12 (*CDK12*) in various cancers and the associated mechanism.

Cancer Type	*CDK12*′s Function	Mechanism	References
Breast cancer (HER2 ^1^ -positive breast cancer)	Tumor promoter	Overexpression of *CDK12* regulates the splicing of ATM ^5^ and DNAJB6-L ^6^ and actives WNT ^7^ and IRS1-ErbB-PI3K ^8^ signaling	[20,59]
Breast cancer (TNBC ^2^)	Tumor suppressor	Loss of *CDK12* leads to downregulation of DDR ^9^ genes	[61,76]
Ovarian cancer (HGSOC ^3^)	Tumor suppressor	Loss of *CDK12* leads to downregulation of DDR ^9^ genes	[21,63,65]
Prostate cancer (mCRPC ^4^)	Tumor suppressor	Loss of *CDK12* leads to downregulation of DDR ^9^ genes	[68,69]
Gastric cancer	Tumor promoter	Overexpression of *CDK12* actives the *CDK12*/CCL21 ^10^ pathway	[75]

^1^ HER2: human epidermal growth factor receptor 2 ^2^ TNBC: triple-negative breast cancer ^3^ HGSOC: high-grade serous ovarian cancer ^4^ mCRPC: metastatic castration-resistant prostate cancer ^5^ ATM: ataxia telangiectasia-mutated ^6^ DNAJB6-L: the long isoform of DNAJB6 (DnaJ homolog subfamily B member 6, MRJ) ^7^ WNT: Wingless-Integrated ^8^ IRS1-ErbB-PI3K: IRS1 (insulin receptor substrate-1)-ErbB (epidermal growth factor receptor)-PI3K (phosphatidylinositol-3-kinase) ^9^ DDR: DNA damage response ^10^ CCL21: CC-chemokine ligand 21.

**Table 2:** *CDK12* as potential target for cancer therapy.

Treatment	Function	Cancer Type	References
Dinaciclib	Inhibition of multiple CDKs including *CDK12*	Breast cancer and metastatic osteosarcoma	[59,96]
THZ1	Inhibition of CDK7/12	Ovarian cancer and neuroblastoma	[67,87]
THZ531	Inhibition of *CDK12*/13	Breast cancer, hepatocellular carcinoma and metastatic osteosarcoma	[56,59,96]
SR-4835	Inhibition of *CDK12*/13	TNBC ^3^ (use with PARP inhibitors)	[76]
PARP ^1^ inhibitors + *CDK12* inhibitors	Synthetic lethality	TNBC ^3^, ovarian cancer and Ewing sarcoma	[76,81,86]
CHK1 ^2^ inhibitors	Synthetic lethality	Ovarian cancer	[79]

^1^ PARP: poly ADP-ribose polymerase ^2^ CHK1: checkpoint kinases 1 ^3^ TNBC: triple-negative breast cancer.
